# Chikungunya Virus E2 N218K Enhances Heparan Sulfate-Dependent Attachment and Viral Internalization While Conferring Partial Attenuation in A129 Mice

**DOI:** 10.3390/pathogens15090993

**Published:** 2026-09-19

**Authors:** Teng Chen, Jie Zhang, Zherui Zhang, Chenglin Deng, Jing Wang, Xin-Can Peng, Yu-Xin Li, Nan Chen, Xing-Yao Huang

**Affiliations:** 1 State Key Laboratory of Pathogen and Biosecurity, Academy of Military Medical Sciences, Beijing 100071, China; chenkuku2024@163.com (T.C.); 18708849270@163.com (J.Z.); 18152670933@163.com (N.C.); 2 Kunming Angel Women and Children’s Hospital, Kunming 650032, China; 3 Key Laboratory of Virology and Biosafety, Wuhan Institute of Virology, Chinese Academy of Sciences, Wuhan 430062, China; 4 University of Chinese Academy of Sciences, Beijing 100049, China

**Keywords:** chikungunya virus, E2 glycoprotein, heparan sulfate, E2 N218K, internalization, attachment, pathogenicity

## Abstract

The E2 glycoprotein of chikungunya virus (CHIKV) binds negatively charged cell-surface heparan sulfate (HS) to facilitate viral attachment and modulate pathogenicity, yet the E2 determinants governing HS-dependent attachment and the effects of altered HS utilization on subsequent infection remain incompletely defined. From a low-passage clinical isolate obtained during the 2010 Guangdong outbreak, we identified a previously unreported E2 substitution, N218K, which introduces a basic residue. In vitro, this mutation enhanced early infection and attachment to mammalian cells and reduced the RNA/PFU ratio; in A129 mice, however, it reduced early serum viral RNA levels, was associated with lower tissue viral RNA burdens at 5 dpi, and delayed mortality, consistent with partial attenuation. Soluble HS competition assays showed that N218K increased HS dependence during viral attachment, whereas DEAE-dextran assays supported a role for charge-dependent interactions in restricting local viral spread. N218K also enhanced viral internalization after normalization of initial attachment, identifying an additional post-attachment phenotype associated with this substitution. Our findings identify N218K as a determinant of HS-dependent attachment and partial attenuation in A129 mice, reveal a “high attachment–low dissemination” phenotype, and further show that this charge-altering E2 substitution enhances post-attachment internalization.

## 1. Introduction

Chikungunya virus (CHIKV) is a re-emerging mosquito-borne alphavirus responsible for repeated large-scale outbreaks worldwide. The 2025 outbreak in Guangdong Province resulted in more than 16,000 locally transmitted cases, including over 10,000 in Foshan, representing the largest documented chikungunya outbreak to date in China [1,2,3]. Infection typically presents with fever, rash, and severe polyarthralgia, and joint symptoms can persist for months to years [4,5,6,7]. Following deposition by infected mosquitoes, CHIKV first replicates at the inoculation site and then spreads through the bloodstream to multiple tissues, infecting diverse cell types including synovial and vascular endothelial cells [7,8,9]. The recent suspension of the live-attenuated vaccine IXCHIQ in the United States over safety concerns underscores the incomplete understanding of the mechanisms linking viral attachment, infection, and attenuation.

Virus entry begins with interactions between the E2 glycoprotein and molecules on the host cell surface [10,11,12,13]. Heparan sulfate (HS) and related sulfated glycosaminoglycans act as attachment factors in this process: their dense negative charge allows electrostatic interaction with basic residues on E2, concentrating virions at the plasma membrane and facilitating infection [13,14,15]. Since E2 covers most of the exposed surface of the alphavirus spike, even single charge-altering substitutions can markedly change HS utilization and viral fitness.

For alphaviruses in general, stronger HS binding tends to raise infectivity in vitro while lowering pathogenicity in vivo. Passaging Sindbis virus in mammalian cells rapidly selects for basic E2 residues that improve HS-dependent attachment [16,17,18]; conversely, variants with weaker HS binding persist longer in blood and reach higher viremia [12]. Similar patterns have been reported for CHIKV. Attenuation of the 181/clone 25 vaccine strain has been linked to E2 substitutions, and residue 82 affects GAG utilization, dissemination, and arthritis in mice [11,13,19]. These observations support an electrostatic trade-off, in which stronger attachment benefits the virus in culture but hampers its spread in the host. Beyond these examples, however, the E2 residues governing HS binding and pathogenicity in CHIKV remain largely unmapped.

Whether changes in HS-dependent attachment also affect subsequent steps of infection is less clear. HS is generally considered an attachment factor that promotes virion association with the cell surface, but alterations in E2 that increase HS dependence may also influence post-attachment processes. In particular, whether such changes affect viral internalization independently of their effect on initial attachment remains poorly understood. Here, plaque analysis and deep sequencing of the low-passage clinical isolate CHIKV GD2010 revealed phenotypic and genetic heterogeneity within the viral population [20]. Through plaque purification, whole-genome sequencing, reverse genetics, cell attachment and infection assays, and infection of A129 mice, we identified E2 residue 218 as a previously unrecognized determinant of HS-dependent attachment in CHIKV [21,22,23]. The N218K substitution enhanced HS-dependent attachment and post-attachment internalization in mammalian cells and was associated with lower early serum viral RNA levels, lower tissue viral RNA burdens at 5 dpi, and delayed mortality in A129 mice. These findings identify E2 N218K as a determinant of HS-dependent attachment and partial attenuation in A129 mice, with an additional effect on post-attachment viral internalization.

## 2. Materials and Methods

### 2.1. Cells and Viruses

Vero (ATCC CCL-81) and A549 (ATCC CCL-185) cells were obtained from the American Type Culture Collection (ATCC, Manassas, VA, USA). C6/36 (ATCC, CRL-1660) cells were obtained from another laboratory. BSR-T7 cells were established and maintained in our laboratory. Vero, A549, and BSR-T7 cells were cultured in Dulbecco’s modified Eagle medium (DMEM; Gibco, Thermo Fisher Scientific, Waltham, MA, USA) supplemented with 10% fetal bovine serum (FBS; Gibco, Waltham, MA, USA) and 1% penicillin–streptomycin at 37 °C in a humidified atmosphere containing 5% CO_2_. C6/36 cells were maintained in RPMI 1640 medium (Gibco) supplemented with 10% FBS (Gibco) and 1% penicillin–streptomycin at 28 °C in a humidified atmosphere containing 5% CO_2_.

The CHIKV GD2010 strain (GenBank accession no. JX088705) [20], isolated from a serum sample collected from a patient in Guangdong, China, had undergone three passages in Vero cells before deep sequencing and plaque purification. For analysis of the GD2010 deep-sequencing data, nucleotide variants present at a frequency of ≥2% were retained. Variant frequencies and corresponding read counts are provided in Table S1. Virus stocks were titrated by plaque assay. CHIKV-L and CHIKV-S were isolated from the heterogeneous GD2010 population by plaque purification as described below. Recombinant viruses were rescued from the corresponding full-length infectious cDNA clones, propagated in Vero cells, and titrated by plaque assay. Virus stocks were aliquoted and stored at −80 °C until use. All experiments involving infectious CHIKV were performed in a certified biosafety level 3 (BSL-3) facility in accordance with institutional and national biosafety guidelines.

### 2.2. Plaque Purification of CHIKV-L and CHIKV-S

The heterogeneous GD2010 virus population was serially diluted and inoculated onto confluent Vero cell monolayers. After adsorption for 1 h at 37 °C, the inoculum was removed, and the cells were overlaid with maintenance medium containing 1% low-melting-point agarose. At 72 h postinfection, individual large and small plaques were separately picked and resuspended in DMEM.

Large- and small-plaque variants were purified through three successive rounds of plaque picking and were designated CHIKV-L and CHIKV-S, respectively. The plaque-purified viruses were propagated in Vero cells, and their complete genome sequences were determined by whole-genome sequencing to confirm the consensus genome sequences of CHIKV-L and CHIKV-S. Whole-genome sequencing confirmed that CHIKV-S and CHIKV-L differed at the four nonsynonymous positions described above, with no additional consensus-level sequence differences detected.

### 2.3. Plaque Assay and Plaque-Size Measurement

Virus samples were serially diluted in DMEM and inoculated onto confluent Vero cell monolayers in 12-well plates. After adsorption for 1 h at 37 °C, the inoculum was removed, and the cells were overlaid with DMEM containing 1% low-melting-point agarose. At 48 h or 72 h postinfection, the cells were fixed with paraformaldehyde and stained with crystal violet. Plaques were photographed, and three plaques were randomly selected from each of three replicate wells for each virus. Plaque diameters were measured using ImageJ version 1.54f. The mean plaque diameter was first calculated for each well from the three measured plaques, and the resulting mean values from the three wells were used for statistical analysis (*n* = 3 wells per virus).

### 2.4. Construction, Rescue, and Confirmation of Recombinant CHIKV Mutants

The full-length infectious CHIKV cDNA clone pACYC-CHIKV [24] was used as the parental backbone. Individual substitutions encoding nsP1-Y157H, nsP1-R171Q, nsP1-P476Q, and E2-N218K were introduced by site-directed mutagenesis using the KLD Enzyme Mix (New England Biolabs, Ipswich, MA, USA). The resulting plasmids were designated rCHIKV-Y157H, rCHIKV-R171Q, rCHIKV-P476Q, and rCHIKV-N218K, respectively. BSR-T7 cells seeded in 12-well plates were transfected with 2 μg of each recombinant plasmid using Lipofectamine 3000 (Thermo Fisher Scientific, Waltham, MA, USA). At 72 h post-transfection, the cells were examined by immunofluorescence assay to confirm successful virus rescue. The rescued viruses were subsequently amplified for two passages in Vero cells. The introduced mutations and the integrity of the rescued viral genomes were confirmed by whole-genome sequencing.

### 2.5. Multistep Growth-Kinetics Assays

Vero cells were infected with CHIKV-L, CHIKV-S, rCHIKV, or rCHIKV-N218K at an MOI of 0.01. After adsorption for 1 h at 37 °C, the inoculum was removed, and the cells were washed twice with PBS before fresh maintenance medium was added. Samples were collected at 0, 12, 24, and 48 h postinfection. At each time point, the entire contents of each well were harvested and subjected to one freeze–thaw cycle. Viral RNA copy numbers were quantified by RT-qPCR, and infectious virus titers were determined by plaque assay where indicated. All experiments were performed in triplicate.

### 2.6. RT-qPCR

Viral RNA was extracted from cell culture samples or supernatants using the PureLink RNA Mini Kit (Thermo Fisher Scientific, Waltham, MA, USA). RT-qPCR was performed on a LightCycler 480 System (Roche Diagnostics, Mannheim, Germany) with One Step PrimeScript RT-PCR Kit (RR064A; TaKaRa Bio Inc., Kusatsu, Japan). The assay targeted the viral nsP1 gene with specific primers (qPCR-F, 5′-TGATCCCGACTCAACCATCCT-3′; qPCR-R, 5′-GGCAAACGCAGTGGTACTTCCT-3′) and a dual-labeled probe (qPCR-Probe, 5′-FAM-TCCGACATCATCCTCCTTGCTGGC-BHQ1-3′) [25].

### 2.7. Immunofluorescent Assay

For immunofluorescence assay (IFA), cells were fixed in cold methanol/acetone (7:3, *v*/*v*) at 12 and 24 h post-infection and incubated with a mouse anti-CHIKV mAb (Cat# M100134, 1:1000 dilution; Zoonogen Co., Ltd., Beijing, China). Following three times of PBS washes, cells were incubated with an Alexa Fluor 488-conjugated secondary antibody (Cat# ab150113, 1:1000 dilution; Abcam, Cambridge, UK) for 1 h at 37 °C. Images were acquired using a fluorescence microscope (Carl Zeiss Microscopy, Oberkochen, Germany).

### 2.8. Virus Attachment Assay

Vero, A549, and C6/36 cells were prechilled at 4 °C and inoculated with CHIKV at an MOI of 1. Virus binding was allowed to proceed for 1 h at 4 °C. The inoculum was then removed, and the cells were washed three times with ice-cold PBS to remove unbound virus. Cell-bound viral RNA was extracted and quantified by RT-qPCR. An aliquot of each corresponding inoculum was retained before infection and quantified by RT-qPCR to determine the input viral RNA copy number. Binding efficiency was calculated as the percentage of input viral RNA that remained cell bound after the attachment step: cell-bound viral RNA/input viral RNA × 100. Experiments were performed in triplicate.

### 2.9. Soluble Heparan Sulfate Competition Assay

Heparan sulfate (HS; Aladdin Scientific, Shanghai, China; catalog no. H304938) was diluted in PBS to the indicated concentrations. CHIKV was mixed with soluble HS and incubated for 30 min at 4 °C. The virus–HS mixtures were then added to prechilled Vero cell monolayers at an MOI of 1 and incubated for 1 h at 4 °C. After removal of the inoculum, the cells were washed with ice-cold PBS and incubated at 37 °C for 24 h in maintenance medium containing 20 mM NH_4_Cl. Virus infectivity was determined by immunofluorescence assay and expressed as the percentage of CHIKV antigen-positive cells.

### 2.10. Heparinase Treatment and Virus-Binding Assay

The heparinase treatment conditions were based on a previously published CHIKV study [15]. Vero cell monolayers were treated with heparinase I/III (Macklin, Shanghai, China; catalog no. H920962) at 1 U/mL for 1 h at 37 °C, using conditions selected based on previously published heparinase treatment protocols. Untreated cells were included as controls. The extent of cell-surface HS depletion was not independently quantified in this study. After treatment, the cells were washed with ice-cold PBS and prechilled at 4 °C. CHIKV was then added at an MOI of 1 and incubated for 1 h at 4 °C. The inoculum was removed, and the cells were washed three times with ice-cold PBS. Cell-bound viral RNA was quantified by RT-qPCR. For each virus, the amount of cell-bound viral RNA after heparinase treatment was normalized to its corresponding untreated control, which was set to 100%, and expressed as the percentage of viral RNA remaining after treatment.

### 2.11. DEAE-Dextran Plaque Assay

The effect of charge-dependent interactions on plaque formation was examined using plaque assays performed with or without DEAE-dextran [26]. Vero cell monolayers were inoculated with serial dilutions of rCHIKV, rCHIKV-N218K, CHIKV-L, or CHIKV-S. After adsorption for 1 h, the inoculum was removed, and cells were overlaid with low-melting-point agarose medium containing either no additive or 150 μg/mL DEAE-dextran (Sigma, St. Louis, MO, USA; catalog no. 80881-1G). Plaques were allowed to develop for 48 h and were then fixed, stained, and imaged. Plaque diameters were measured and analyzed as described in the plaque assay and plaque-size measurement section.

### 2.12. E2 Sequence Alignment and Conservation Analysis

Full-length E2 amino acid sequences representing the Asian, East/Central/South African (ECSA), ECSA–Indian Ocean lineage (ECSA-IOL), ECSA-2, and West African lineages of CHIKV were retrieved from GenBank. SYD2025 was included as the representative strain of the ECSA-2 lineage. Full-length E2 sequences of representative arthritogenic alphaviruses, including Mayaro virus (MAYV), Getah virus (GETV), Ross River virus (RRV), O’nyong-nyong virus (ONNV), and CHIKV, were also included for interspecies comparison. Sequences were aligned using MAFFT version 7 [27] with default parameters. Conservation of CHIKV E2 residue N218 and the corresponding residues in related alphaviruses was assessed from the full-length E2 alignments. For visualization, only the region corresponding to CHIKV E2 residues 180–240 was displayed using ESPript version 3, and CHIKV residue N218 and the corresponding conserved asparagine residues were highlighted with yellow boxes.

### 2.13. Mouse Experiments

Four-week-old A129 mice were assigned to experimental groups according to body weight to ensure comparable baseline body weights among groups. For both animal experiments, mice were inoculated subcutaneously in the hind footpad with 20 PFU of virus in a total volume of 20 μL. The comparisons included parental rCHIKV versus rCHIKV-N218K and CHIKV-L versus CHIKV-S.

For each comparison, eight mice were included per group. Five mice per group were monitored for survival, body weight, and foot swelling, while three additional mice per group were used for virological analyses. Body weight and foot swelling were recorded daily. Body weight was expressed as a percentage of the value measured on the day of inoculation. Foot swelling was measured using a digital caliper and expressed as the product of foot width and thickness (mm^2^). Animals were monitored until 9 days postinfection (dpi) or until predefined humane endpoints were reached.

Mice were anesthetized with sodium pentobarbital before blood collection. Blood samples were collected from the retro-orbital venous plexus at 1 and 3 dpi. Serum was separated by centrifugation at 8000 rpm for 5 min and stored at −80 °C until analysis. At 5 dpi, mice assigned to virological analyses were euthanized by an intraperitoneal overdose of sodium pentobarbital, and the heart, liver, spleen, lung, kidney, brain, gastrocnemius muscle from the inoculated hind limb, ankle joint, and inoculated footpad were collected. Tissue samples were immediately stored at −80 °C until RNA extraction.

Viruses recovered from joint tissues collected at 5 dpi were subjected to plaque assay and sequence analysis of the E2-218 region to assess genetic and phenotypic stability in vivo.

Each animal study was performed once as an independent experiment.

### 2.14. Virus Internalization Assay

Vero, A549, and C6/36 cells were prechilled at 4 °C before virus inoculation. The inputs of rCHIKV and rCHIKV-N218K were adjusted separately on the basis of viral RNA copy number to obtain comparable levels of initially cell-bound viral RNA after attachment. Viruses were allowed to attach for 1 h at 4 °C, after which unbound virus was removed by washing with ice-cold PBS. An aliquot of cells was collected to quantify the initially cell-bound viral RNA by RT-qPCR. The remaining cells were shifted to 37 °C to allow viral internalization. Surface-associated virus was subsequently stripped by treatment with 0.1 M glycine-HCl (pH 2.5) for 2 min at room temperature, and the remaining glycine-resistant cell-associated viral RNA was quantified by RT-qPCR as a measure of viral internalization. Internalization efficiency was calculated as follows:

Internalization efficiency (%) = glycine-resistant viral RNA/initially cell-bound viral RNA × 100.

### 2.15. Animal Ethics Statement

All mice were cared for in accordance with the recommendations in the Guide for the Care and Use of Laboratory Animals [28]. All animal experiments were conducted in an animal biosafety level 3 (ABSL-3) facility at the Wuhan Institute of Virology, Chinese Academy of Sciences, under a protocol approved by the Laboratory Animal Ethics Committee of the Wuhan Institute of Virology, Chinese Academy of Sciences (permit no. WIVA26202401).

### 2.16. Statistical Analysis

Statistical analyses were performed using GraphPad Prism version 8. Data are presented as the mean ± standard deviation (SD) unless otherwise indicated. The numbers of biological replicates or animals included in each analysis are specified in the corresponding figure legends.

Comparisons between two groups were performed using an unpaired two-tailed Student’s *t* test. Time-course data, including viral growth kinetics, body weight, and foot swelling, were analyzed using two-way analysis of variance (ANOVA) followed by Šídák’s multiple-comparisons test. Soluble HS competition data were analyzed using two-way ANOVA followed by Šídák’s multiple-comparisons test, with virus and HS concentration included as the two factors. Survival curves were compared using the log-rank (Mantel–Cox) test.

Viral RNA loads in serum and tissues were analyzed using two-way ANOVA followed by Šídák’s multiple-comparisons test, with virus and sampling site/tissue included as the two factors, as appropriate. A *p* value of <0.05 was considered statistically significant. Significance is indicated as follows: *, *p* < 0.05; **, *p* < 0.01; ***, *p* < 0.001; ****, *p* < 0.0001; ns, not significant.

## 3. Results

### 3.1. Phenotypic and Genetic Heterogeneity in GD2010 Reveals a Stable Small-Plaque Variant with Enhanced Attachment to Mammalian Cells

**Figure 1:**
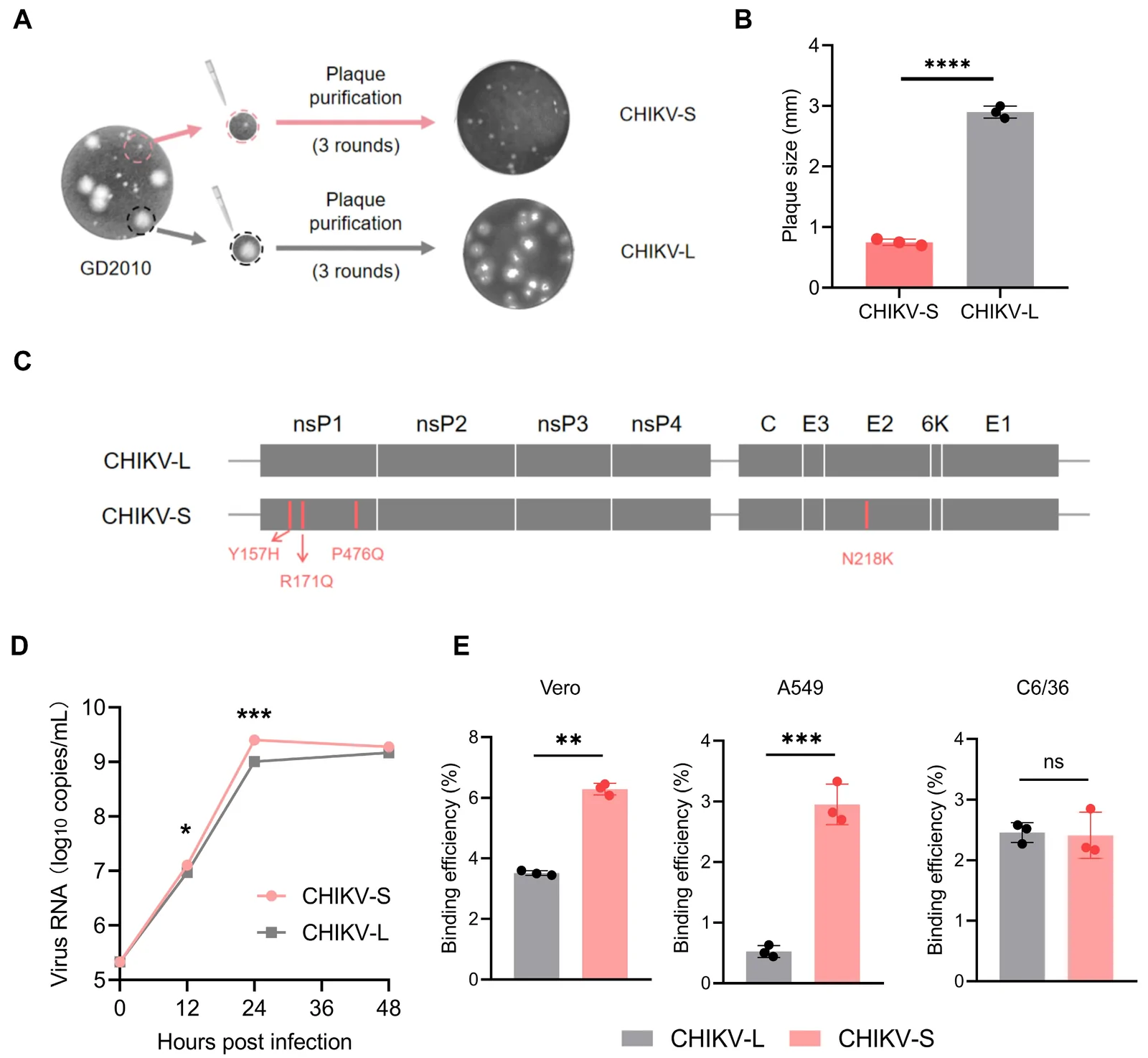
Plaque heterogeneity in the GD2010 isolate reveals a stable small-plaque variant with four candidate amino acid substitutions and an early growth advantage in Vero cells. (**A**) Schematic representation of the isolation of small-plaque CHIKV-S and large-plaque CHIKV-L from the heterogeneous GD2010 isolate by three rounds of plaque purification in Vero cells. Representative plaque images are shown. (**B**) Plaque diameters of CHIKV-S and CHIKV-L in Vero cells at 72 h postinfection. Data are presented as the mean ± SD (*n* = 3 wells). (**C**) Schematic representation of the CHIKV genome showing the four candidate amino acid substitutions associated with CHIKV-S: nsP1-Y157H, nsP1-R171Q, nsP1-P476Q, and E2-N218K. (**D**) Multistep growth kinetics of CHIKV-S and CHIKV-L in Vero cells. Cells were infected at an MOI of 0.01, and viral RNA copies were quantified by qRT-PCR at the indicated times. (**E**) Cell-surface binding efficiencies of CHIKV-L and CHIKV-S in Vero, A549, and C6/36 cells. Cells were inoculated with each virus at an MOI of 1 at 4 °C, and binding efficiency was determined by quantifying cell-bound viral RNA. Data are presented as the mean ± SD from three independent experiments. Statistical significance was determined using an unpaired two-tailed Student’s *t* test in panels (**B**,**E**) and two-way ANOVA in panel (**D**). *, *p* < 0.05; **, *p* < 0.01; ***, *p* < 0.001; ****, *p* < 0.0001; ns, not significant.

The low-passage clinical isolate GD2010 (GenBank accession no. JX088705.1), obtained during the 2010 chikungunya outbreak in Guangdong, China, produced distinct large- and small-plaque phenotypes (Figure 1A). Deep sequencing of the GD2010 virus population identified multiple nucleotide variants, including four nonsynonymous substitutions: nsP1 Y157H (60.36%; depth, 11,723 reads), nsP1 R171Q (10.89%; depth, 11,641 reads), nsP1 P476Q (23.23%; depth, 7076 reads), and E2 N218K (36.88%; depth, 14,185 reads) (Table S1).

Plaque purification yielded two variants, designated CHIKV-L and CHIKV-S, with large- and small-plaque phenotypes, respectively (Figure 1A,B). Whole-genome sequencing showed that CHIKV-S differed from CHIKV-L at four amino acid positions: nsP1 Y157H, R171Q, and P476Q and E2 N218K (Figure 1C). Because changes in E2 surface charge are known to influence CHIKV plaque phenotype, cell attachment, replication, and pathogenicity [19,29], N218K—which replaces a neutral asparagine with a positively charged lysine—was prioritized for investigation in this study, as the functions of the other three substitutions have been reported previously or are being investigated separately [30,31].

Multistep growth analysis in Vero cells showed that CHIKV-S reached higher viral RNA levels than CHIKV-L at 12 and 24 h postinfection, whereas the two viruses were comparable at 48 h (Figure 1D). During mixed serial passage in Vero cells, only the small-plaque phenotype was detectable after five passages (Figure S1), indicating strong enrichment of CHIKV-S under these culture conditions. CHIKV-S also maintained its small-plaque phenotype during continued passage.

We next compared the attachment efficiencies of CHIKV-L and CHIKV-S in Vero, A549, and C6/36 cells. CHIKV-S attached significantly more efficiently than CHIKV-L in both Vero (6.27 ± 0.26% vs. 3.48 ± 0.04%) and A549 cells (2.95 ± 0.33% vs. 0.52 ± 0.10%) (Figure 1E). In contrast, the two viruses did not differ in C6/36 mosquito cells (2.47 ± 0.14% vs. 2.51 ± 0.37%). Thus, CHIKV-S shows enhanced attachment to mammalian cells and a modest early growth advantage in Vero cells.

### 3.2. CHIKV-S Exhibits Partial Attenuation and Reduced Systemic Viral RNA Burdens in A129 Mice

**Figure 2:**
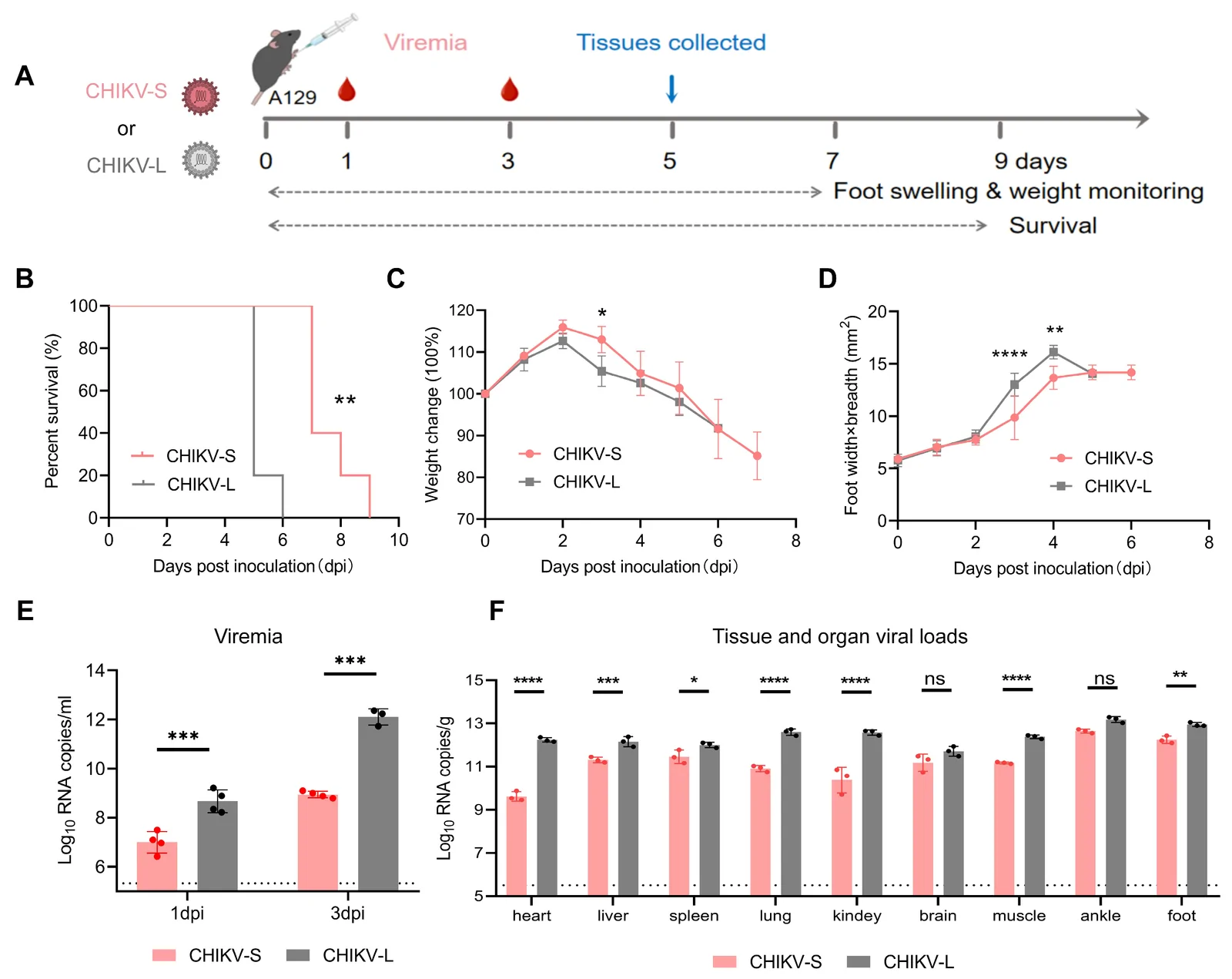
CHIKV-S is attenuated in A129 mice and shows reduced systemic dissemination compared with CHIKV-L. (**A**) Experimental design. Four-week-old A129 mice were inoculated subcutaneously in the hind footpad with 20 PFU of CHIKV-S or CHIKV-L in a volume of 20 μL. Eight mice were included in each group. Five mice per group were monitored for survival, body weight, and footpad swelling. Serum samples were collected from four of these five mice at 1 and 3 days post-inoculation (dpi) for viremia analysis. The remaining three mice were used for tissue collection at 5 dpi. (**B**) Survival of mice infected with CHIKV-S or CHIKV-L. (**C**) Changes in body weight after infection, expressed as a percentage of the body weight recorded on the day of inoculation. (**D**) Swelling of the inoculated footpad, calculated as the product of two perpendicular footpad measurements. (**E**) Viral RNA loads in serum collected at 1 and 3 dpi, as determined by RT-qPCR. (**F**) Viral RNA loads in tissues collected at 5 dpi. Heart, liver, spleen, lung, kidney, brain, gastrocnemius muscle from the inoculated hind limb, ankle joint, and foot tissue after removal of the skin were collected and homogenized. Viral RNA loads were normalized to tissue weight and are expressed as RNA copies per gram of tissue. Dotted lines indicate the limits of detection. Data are from one independent animal experiment. Data are presented as the mean ± SD. Survival curves were compared using the log-rank test. Data in panels (**C**–**F**) were analyzed using two-way ANOVA followed by Šídák’s multiple-comparisons test. *, *p* < 0.05; **, *p* < 0.01; ***, *p* < 0.001; ****, *p* < 0.0001; ns, not significant.

Although CHIKV-S showed a modest early growth advantage in Vero cells, whether this translated into altered virulence in vivo was unknown. To address this, A129 mice were inoculated subcutaneously in the hind footpad with 20 PFU of either CHIKV-S or CHIKV-L (Figure 2A).

CHIKV-L-infected mice began to die at 5 days post-inoculation (dpi), and all animals had died by 6 dpi (5/5). In contrast, mortality in the CHIKV-S group was delayed, with death beginning at 7 dpi and all animals succumbing by 9 dpi (5/5) (Figure 2B). Both groups lost weight progressively, but CHIKV-S-infected mice showed significantly less weight loss at 3 dpi (Figure 2C). Foot swelling developed in both groups but was less pronounced in CHIKV-S-infected mice at 3 and 4 dpi (Figure 2D).

Serum viral RNA levels were significantly lower in CHIKV-S-infected mice than in CHIKV-L-infected mice at both 1 and 3 dpi (Figure 2E). At 5 dpi, viral RNA was detected in all tissues examined from both groups. However, CHIKV-S produced significantly lower viral RNA levels in the heart, liver, spleen, lung, kidney, gastrocnemius muscle, and foot, whereas the two viruses did not differ in the brain or ankle (Figure 2F). CHIKV-S thus disseminates less efficiently and is partially attenuated in A129 mice.

### 3.3. E2 N218K Is Sufficient to Confer the Small-Plaque Phenotype

**Figure 3:**
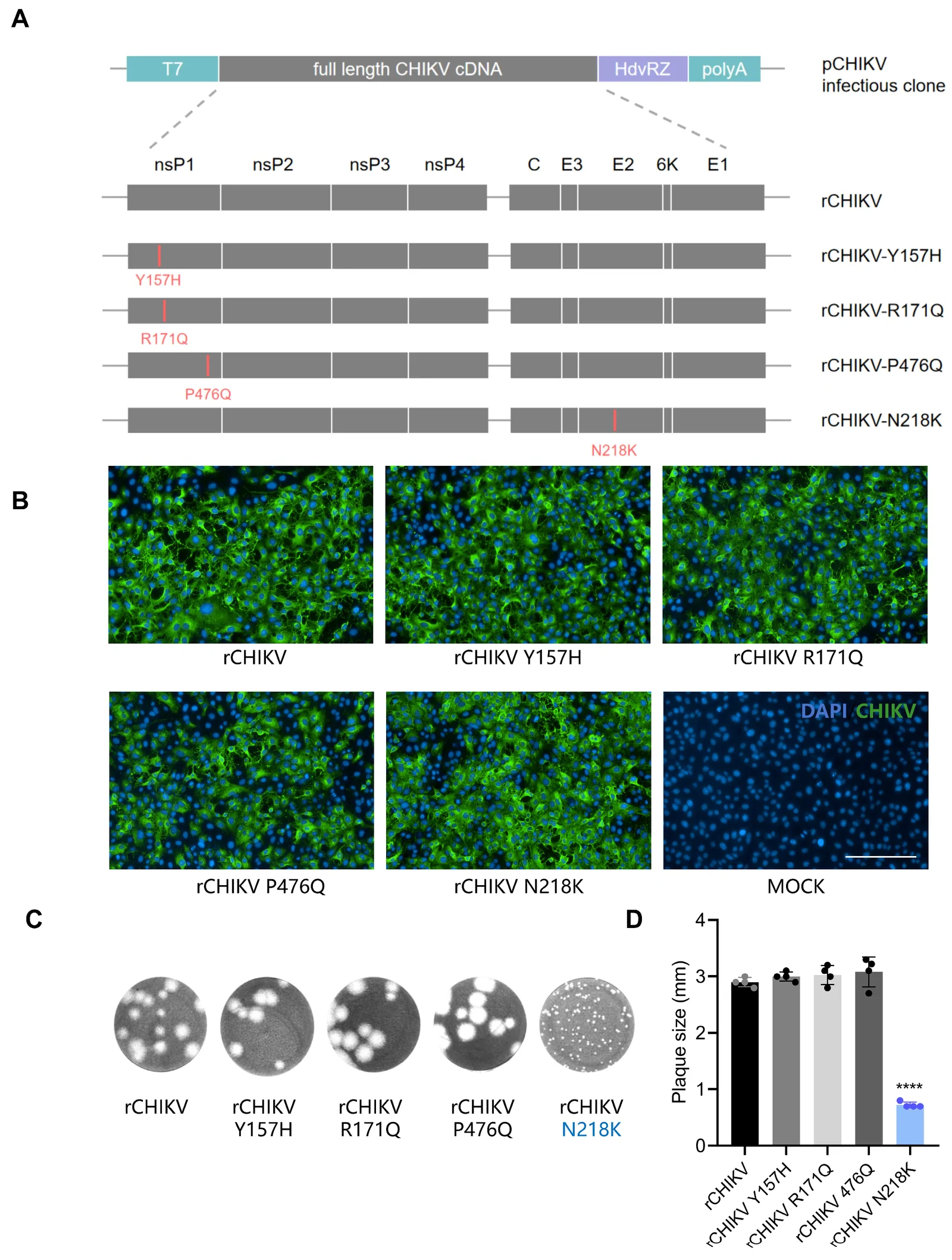
Reverse genetics identifies the E2-N218K substitution as the critical determinant of the small-plaque phenotype. (**A**) Schematic of the parental CHIKV infectious clone, pCHIKV, and the four single-amino-acid mutant viruses. The Y157H, R171Q, and P476Q substitutions in nsP1 and the N218K substitution in E2 were individually introduced into the parental infectious clone to generate rCHIKV Y157H, rCHIKV R171Q, rCHIKV P476Q, and rCHIKV 218K, respectively. The infectious clone contained a T7 promoter, the full-length CHIKV cDNA, a poly(A) tract, and a hepatitis delta virus ribozyme. (**B**) Representative immunofluorescence images confirming the recovery of rCHIKV and the four mutant viruses following transfection of sequence-verified plasmids into BSR-T7 cells. Viral antigen is shown in green, and nuclei are shown in blue. Mock-transfected cells were included as a negative control. Scale bar, 200 μm. (**C**) Representative plaque morphologies of rCHIKV and the four single-amino-acid mutant viruses at 72 h postinfection. (**D**) Quantification of plaque diameters. Data are presented as the mean ± SD (*n* = 3 wells). Statistical significance was determined by one-way ANOVA followed by Dunnett’s multiple-comparisons test, with each mutant compared with rCHIKV. ****, *p* < 0.0001.

To determine which of the four amino acid substitutions identified in CHIKV-S contributed to the small-plaque phenotype, we used a full-length infectious cDNA clone of CHIKV (pCHIKV) [24] to generate four recombinant viruses, each carrying one of the amino acid substitutions identified in CHIKV-S (Figure 3A). The parental infectious clone and CHIKV-L were identical at the four candidate positions examined (nsP1 Y157, R171, P476, and E2 N218), allowing the effect of each substitution to be evaluated individually in the parental infectious-clone background. Following transfection into BSR-T7 cells, the parental virus and all four mutant viruses were successfully rescued, as confirmed by viral antigen expression (Figure 3B).

Plaque assays in Vero cells showed that the three nsP1 mutants, rCHIKV-Y157H, rCHIKV-R171Q, and rCHIKV-P476Q, retained large-plaque morphologies comparable to that of the parental rCHIKV. In contrast, rCHIKV-N218K formed significantly smaller plaques (*p* < 0.0001), closely reproducing the small-plaque phenotype of CHIKV-S (Figure 3C,D). These results demonstrate that, among the four substitutions tested individually, E2 N218K alone was sufficient to confer the small-plaque phenotype.

### 3.4. E2 N218K Enhances Attachment to Mammalian Cells and Increases Apparent Infectivity

**Figure 4:**
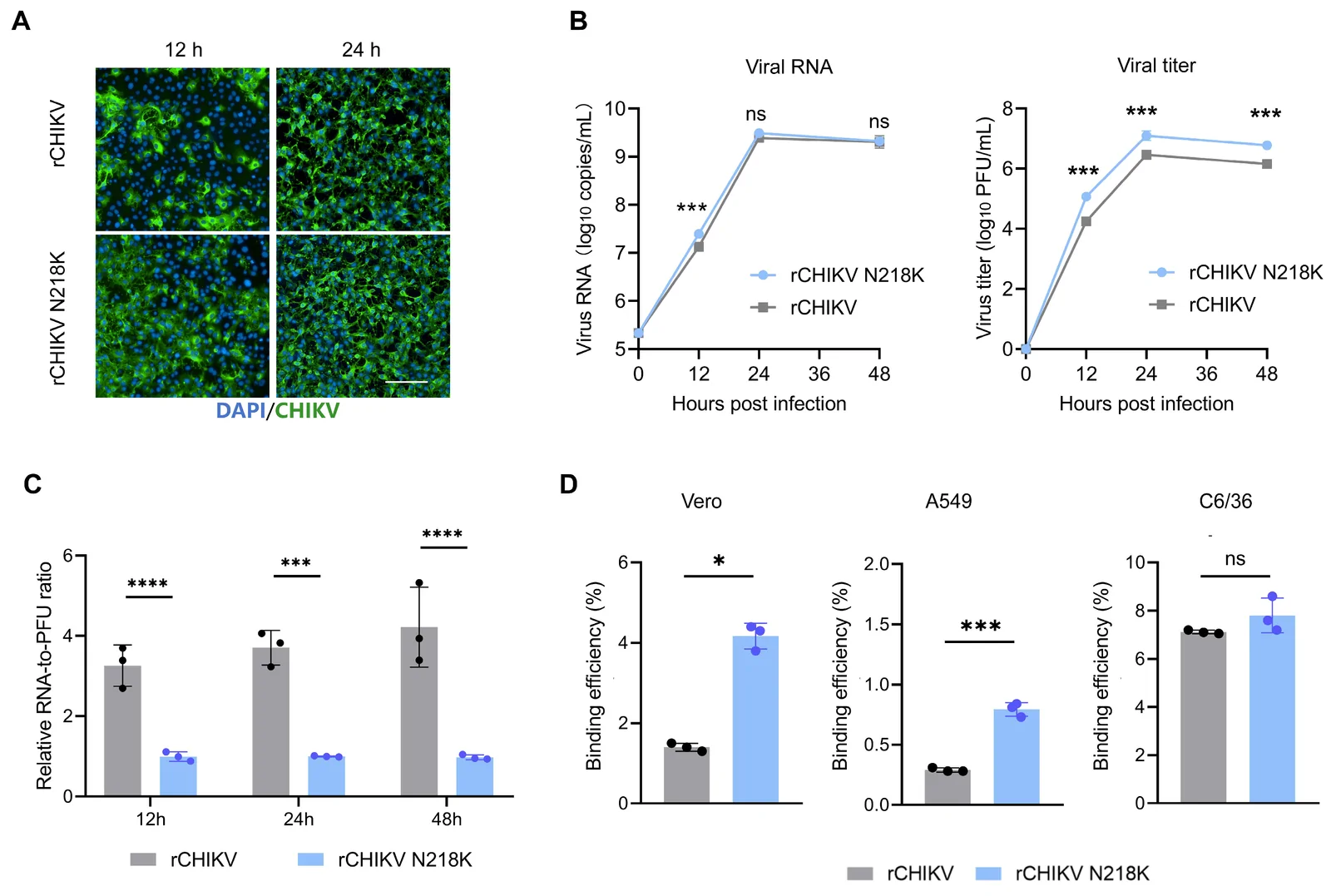
The E2 N218K substitution enhances mammalian-cell attachment in association with increased apparent infectivity. (**A**) Representative immunofluorescence images of Vero cells infected with rCHIKV or rCHIKV-N218K at a multiplicity of infection (MOI) of 0.01. Cells were fixed at 12 and 24 h postinfection. Viral antigen is shown in green, and nuclei are shown in blue. Scale bar, 200 μm. (**B**) Multistep growth kinetics of rCHIKV and rCHIKV-N218K in Vero cells. Cells were infected at an MOI of 0.01, and culture supernatants were collected at the indicated times postinfection. Viral RNA copy numbers were measured by RT-qPCR, and infectious virus titers were determined by plaque assay. (**C**) Relative viral RNA-to-PFU ratios in culture supernatants collected at 12, 24, and 48 h postinfection. The viral RNA-to-PFU ratio was calculated for each sample and normalized to the mean value for rCHIKV-N218K at the corresponding time point. (**D**) Binding efficiencies of rCHIKV and rCHIKV-N218K in Vero, A549, and C6/36 cells. Binding efficiency was expressed as the percentage of input viral RNA that remained cell bound after the binding step. Data are presented as the mean ± SD from three independent experiments. Data in panels (**B**,**C**) were analyzed by two-way ANOVA followed by multiple-comparisons testing. Comparisons in panel (**D**) were performed using unpaired two-tailed Student’s *t* tests. *, *p* < 0.05; ***, *p* < 0.001; ****, *p* < 0.0001; ns, not significant.

We next examined whether N218K alone could reproduce the growth characteristics of CHIKV-S. Vero cells were infected with rCHIKV or rCHIKV-N218K at an MOI of 0.01. More viral antigen-positive cells were detected in rCHIKV-N218K-infected cultures at 12 h postinfection, whereas the difference was less apparent at 24 h (Figure 4A). Viral RNA levels were also higher for rCHIKV-N218K at 12 h but were comparable between the two viruses at 24 and 48 h. In contrast, rCHIKV-N218K produced significantly higher infectious titers at all three time points (Figure 4B). The viral RNA-to-PFU ratio of parental rCHIKV was approximately three- to fourfold higher than that of rCHIKV-N218K (Figure 4C), indicating greater apparent infectivity of the N218K mutant relative to the amount of measured viral RNA.

Since E2 mediates virus attachment, we examined whether this phenotype correlated with altered attachment to host cells. Attachment efficiency increased from approximately 1.4% for rCHIKV to 4.1% for rCHIKV-N218K in Vero cells and from approximately 0.3% to 0.8% in A549 cells. In contrast, attachment was comparable between rCHIKV and rCHIKV-N218K in C6/36 cells (approximately 7.1% and 8.1%, respectively) (Figure 4D). Thus, N218K enhances early infection and lowers the viral RNA-to-PFU ratio, and increases virus attachment to mammalian cells.

### 3.5. E2 N218K Reduces Systemic Viral RNA Burdens and Confers Partial Attenuation in A129 Mice

Because N218K enhanced HS-dependent attachment in mammalian cells but restricted plaque spread, we next examined its effect in vivo. Four-week-old A129 mice were infected with parental rCHIKV or rCHIKV-N218K.

**Figure 5:**
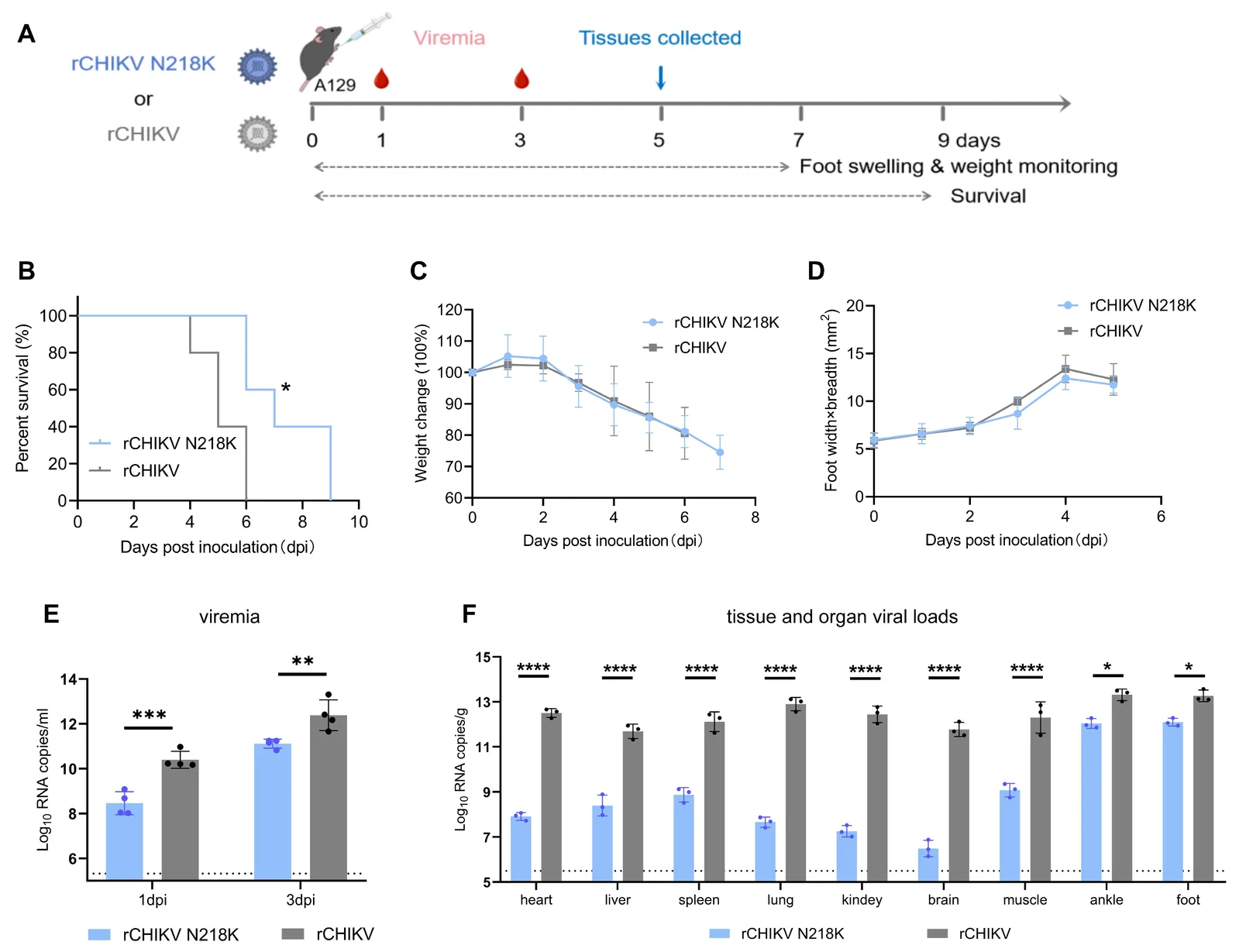
The E2 N218K substitution delays mortality and reduces systemic viral RNA loads in A129 mice. (**A**) Experimental design. Four-week-old A129 mice were inoculated subcutaneously in the hind footpad with 20 PFU of rCHIKV or rCHIKV-N218K. Eight mice were included in each group. Five mice per group were monitored for survival, body weight, and footpad swelling, whereas the remaining three mice were used for serum collection at 1 and 3 days post inoculation (dpi) and tissue collection at 5 dpi. (**B**) Survival of mice infected with rCHIKV or rCHIKV-N218K. Survival curves were compared using the log-rank (Mantel–Cox) test. (**C**) Changes in body weight after infection, expressed as a percentage of the body weight recorded on the day of inoculation. (**D**) Swelling of the inoculated footpad, calculated as the product of two perpendicular footpad measurements. (**E**) Viral RNA loads in serum collected at 1 and 3 dpi, as determined by RT-qPCR. (**F**) Viral RNA loads in tissues collected at 5 dpi. Heart, liver, spleen, lung, kidney, brain, gastrocnemius muscle from the inoculated hind limb, ankle joint, and foot tissue were collected and homogenized. Viral RNA loads were normalized to tissue weight and are expressed as RNA copies per gram of tissue. Dotted lines indicate the limits of detection. Data are from one independent animal experiment. Data are presented as the mean ± SD. Data in panels (**C**–**F**) were analyzed using two-way ANOVA followed by Šídák’s multiple-comparisons test. *, *p* < 0.05; **, *p* < 0.01; ***, *p* < 0.001; ****, *p* < 0.0001.

Mice were inoculated subcutaneously in the hind footpad with 20 PFU of virus in 20 μL (Figure 5A). Both viruses caused lethal infection, but death was delayed in the rCHIKV-N218K group. Mice infected with parental rCHIKV began to die at 4 days post inoculation (dpi), and all had died by 6 dpi (5/5). In the rCHIKV-N218K group, deaths began at 6 dpi and continued through 9 dpi (5/5), resulting in significantly longer survival (Figure 5B). Body-weight loss was similar between the two groups (Figure 5C). Footpad swelling was generally lower in rCHIKV-N218K-infected mice, although the difference did not reach statistical significance (Figure 5D).

Serum viral RNA levels were significantly lower in the rCHIKV-N218K group at both 1 and 3 dpi, indicating reduced early viremia (Figure 5E). At 5 dpi, viral RNA was detected in all tissues examined in both groups. However, viral RNA levels were significantly lower in rCHIKV-N218K-infected mice in the heart, liver, spleen, lung, kidney, brain, skeletal muscle, ankle joint, and inoculated foot (Figure 5F). Because tissue samples were collected at 5 dpi, when disease progression had already begun to differ between the two groups, the tissue viral RNA levels should be interpreted together with the earlier serum data.

To assess the in vivo stability of N218K, viruses recovered from joint tissues at 5 dpi were further analyzed by sequencing and plaque assay. Sequence analysis confirmed retention of the expected E2-218 residue in both groups, with sequencing depths of 1062 reads for rCHIKV and 1309 reads for rCHIKV-N218K. No mixed sequence or K-to-N reversion was detected in the N218K group. Consistently, recovered rCHIKV-N218K retained a uniform small-plaque phenotype, whereas parental rCHIKV retained the large-plaque phenotype (Figure S2). These results indicate that the N218K genotype and associated plaque phenotype remained stable through 5 dpi in vivo.

These results support reduced systemic dissemination and partial attenuation of rCHIKV-N218K in A129 mice, as reflected by lower early viremia, reduced tissue viral RNA levels, and delayed mortality.

### 3.6. E2 N218K Enhances Heparan Sulfate-Dependent Attachment and Restricts Local Viral Spread Through Charge-Dependent Interactions

N218K replaces a neutral asparagine with a positively charged lysine and selectively enhances virus binding to mammalian cells. Because CHIKV can use cell-surface heparan sulfate (HS) for attachment, we examined whether the increased mammalian-cell binding associated with N218K resulted from greater HS dependence and whether charge-dependent interactions contributed to its restricted local spread [32,33,34].

To address these questions, we used three complementary approaches—soluble HS competition, enzymatic reduction of cell-surface HS, and extracellular charge modulation. The first two approaches were used to assess the contribution of HS to viral attachment, whereas the charge-modulation experiment was used to determine whether electrostatic interactions contributed to the small-plaque phenotype.

**Figure 6:**
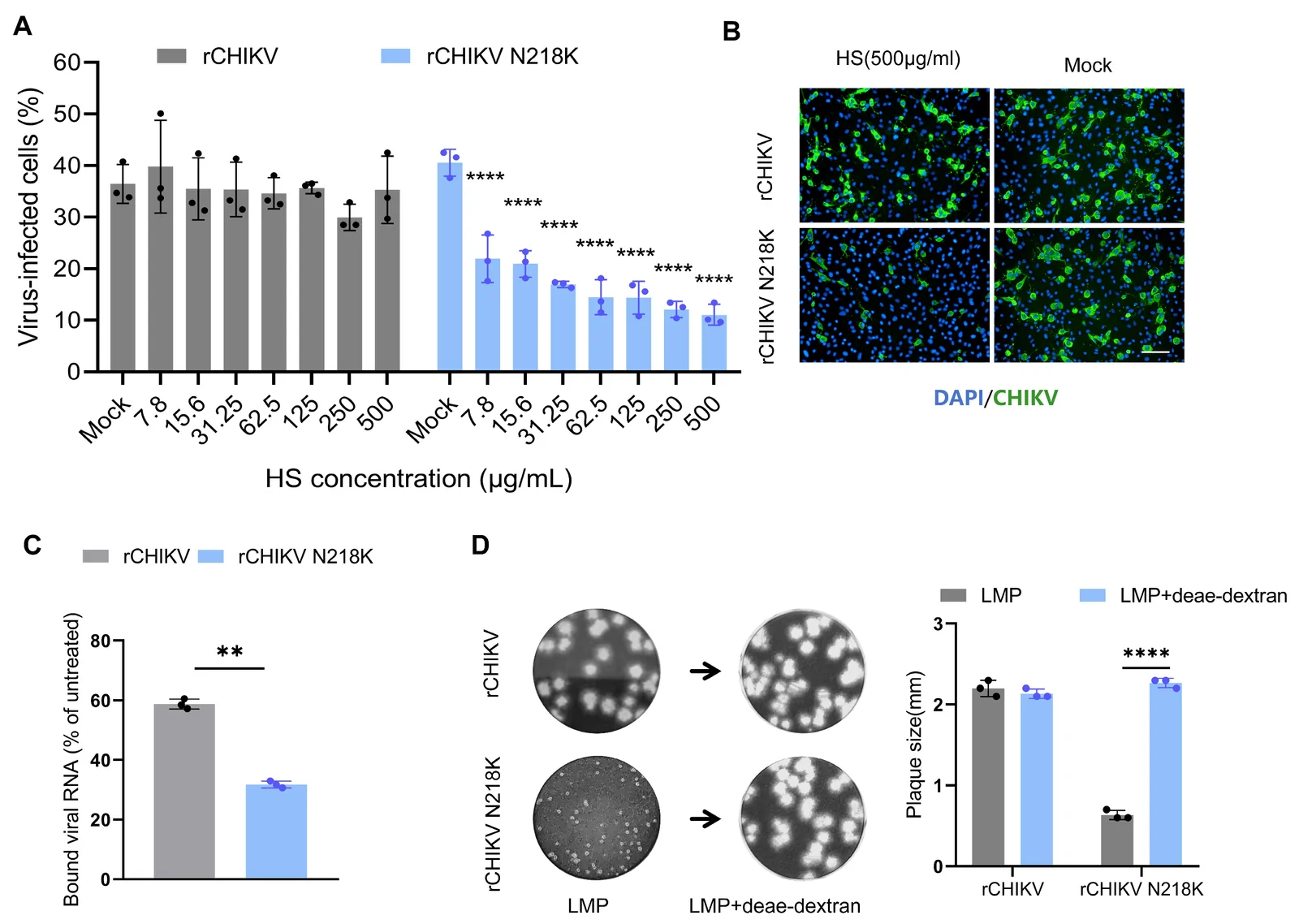
E2 N218K enhances heparan sulfate-dependent attachment and confers a DEAE-dextran-reversible small-plaque phenotype. (**A**,**B**) Inhibition of infection by soluble heparan sulfate (HS). rCHIKV and rCHIKV-N218K were preincubated with the indicated concentrations of soluble HS at 4 °C and then added to Vero cells and allowed to bind at 4 °C. After the binding step, the cells were shifted to 37 °C and cultured for 24 h in the presence of NH_4_Cl. The percentage of infected cells was quantified by immunofluorescence microscopy (**A**). Representative images of cells infected in the absence of HS or in the presence of 500 μg/mL HS are shown in panel (**B**). Viral antigen is shown in green, and nuclei are shown in blue. Scale bar, 200 μm. (**C**) Effect of cell-surface HS removal on virus binding. Vero cells were treated with heparinase I/III at 1 U/mL for 1 h at 37 °C or left untreated. After removal of the enzyme, rCHIKV or rCHIKV-N218K was added to the cells and allowed to bind for 1 h at 4 °C. Cell-bound viral RNA was quantified by RT-qPCR and expressed as a percentage of the corresponding untreated control. (**D**) Effect of DEAE-dextran on plaque morphology. Plaque assays were performed using a standard low-melting-point (LMP) agarose overlay or an LMP overlay supplemented with DEAE-dextran. Representative plaque morphologies are shown on the left, and plaque diameters are quantified on the right. DEAE-dextran restored the plaque diameter of rCHIKV-N218K to a level similar to that of parental rCHIKV. Data are presented as the mean ± SD (*n* = 3 wells). Panel (**A**) was analyzed by two-way ANOVA with comparisons between each HS concentration and the corresponding no-HS control. Panel (**C**) was analyzed using an unpaired two-tailed Student’s *t* test. Panel (**D**) was analyzed by two-way ANOVA with comparisons between the two overlay conditions within each virus. **, *p* < 0.01; ****, *p* < 0.0001.

Preincubation with soluble HS had little effect on parental rCHIKV over the concentration range of 7.8–500 μg/mL. The proportion of infected cells was 36.47% in the mock-treated group and ranged from 29.97% to 39.80% after HS treatment, with no significant differences from the mock control. In contrast, rCHIKV-N218K was strongly inhibited by soluble HS. The proportion of infected cells decreased from 40.57% in the mock-treated group to 21.97% at 7.8 μg/mL HS and declined further as the HS concentration increased, reaching 11.10% at 500 μg/mL (Figure 6A,B). Thus, N218K markedly increased sensitivity to soluble HS competition.

We next reduced cell-surface HS by treating Vero cells with heparinase I/III before virus binding. Heparinase treatment decreased cell-bound viral RNA for both viruses, but the reduction was greater for rCHIKV-N218K. Relative to untreated cells, bound viral RNA was reduced to 59.48% for parental rCHIKV and to 31.14% for rCHIKV-N218K (Figure 6C). Together with the soluble HS competition results, these data indicate that N218K increases the dependence of virus attachment on cell-surface HS.

We then examined whether charge-dependent interactions contributed to the small-plaque phenotype. Following virus inoculation, cells were overlaid with low-melting-point agarose containing the polycation DEAE-dextran at 150 μg/mL, and plaque size was assessed at 48 h postinfection. DEAE-dextran had little effect on the plaque size of parental rCHIKV but markedly increased the plaque size of rCHIKV-N218K to a level comparable to that of the parental virus (Figure 6D).

This preferential restoration of the N218K plaque phenotype supports a role for charge-dependent extracellular interactions in restricting local viral spread. Although DEAE-dextran is not specific for HS, when considered together with the soluble HS competition and heparinase results, the combined data support a model in which increased HS-dependent electrostatic interactions contribute to the restricted spread and small-plaque phenotype of rCHIKV-N218K.

Together, these findings show that E2 N218K increases HS-dependent attachment to mammalian cells and support a model in which enhanced HS-dependent, charge-mediated interactions contribute to restricted local viral spread.

### 3.7. N218K Enhances Viral Internalization in Mammalian Cells After Normalization of Initial Attachment

**Figure 7:**
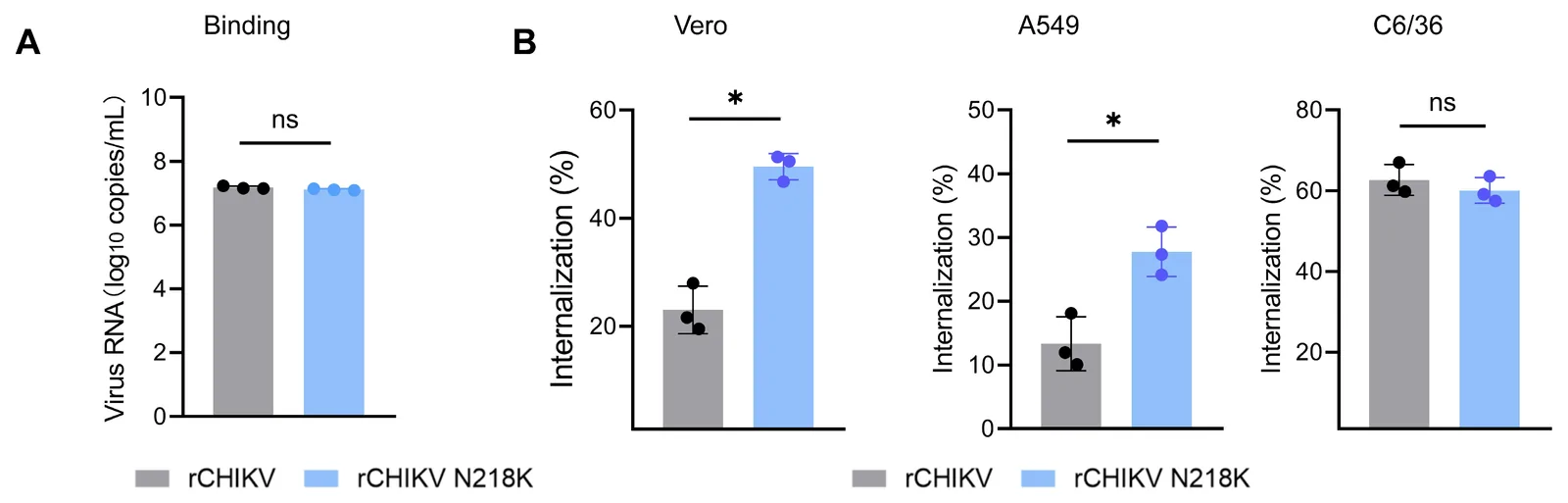
E2 N218K enhances viral internalization after normalization of initial binding. (**A**) Comparable levels of cell-bound viral RNA were established for rCHIKV and rCHIKV-N218K before the internalization assay to control for differences in viral binding. Viral RNA was quantified by RT-qPCR. (**B**) Internalization efficiencies of rCHIKV and rCHIKV-N218K in Vero, A549, and C6/36 cells. After establishing comparable amounts of cell-bound virus, cells were shifted to 37 °C to allow viral internalization. Residual surface-bound virions were removed by glycine wash, and internalized viral RNA was quantified by RT-qPCR. Internalization efficiency was calculated relative to the amount of initially cell-bound viral RNA. Data are presented as mean ± SD. * *p* < 0.05; ns, not significant.

Because N218K enhanced attachment to mammalian cells, we next examined whether this substitution also affected viral internalization after the initial attachment step. To distinguish the effect on internalization from that on attachment, the inputs of rCHIKV and rCHIKV-N218K were adjusted separately on the basis of viral RNA copy number to obtain comparable amounts of cell-bound rCHIKV and rCHIKV-N218K before internalization. No significant difference in cell-associated viral RNA was detected between the two viruses under these conditions (Figure 7A). The cells were then shifted to 37 °C to allow internalization, followed by glycine washing to strip surface-associated virus. Glycine-resistant cell-associated viral RNA was quantified by RT-qPCR as a measure of viral internalization.

With comparable amounts of virus initially bound to the cell surface, internalization efficiency increased from 23.02% for rCHIKV to 49.55% for rCHIKV-N218K in Vero cells, and from 13.35% to 27.75% in A549 cells. No significant difference was observed in C6/36 cells (Figure 7B). Thus, N218K enhanced viral internalization in mammalian cells independently of its effect on the initial level of attachment. These results identify enhanced post-attachment internalization as an additional phenotype associated with N218K; the receptor or cellular mechanism responsible for this effect remains to be determined.

## 4. Discussion

From the low-passage clinical isolate GD2010 obtained during the 2010 Guangdong outbreak, we identified a previously unreported E2 substitution, N218K, which introduces a basic lysine residue. Reverse-genetic analysis showed that N218K alone enhanced early infection, infectious virus production, and attachment to mammalian cells while reducing the viral RNA-to-PFU ratio. In A129 mice, however, N218K reduced early viremia and tissue viral RNA levels and delayed mortality. Soluble HS competition and heparinase treatment showed that N218K increased HS dependence, whereas DEAE-dextran treatment linked charge-dependent interactions to restricted local viral spread. N218K also enhanced viral internalization after controlling for initial attachment, indicating that the effect of N218K extends beyond initial attachment to a post-attachment internalization step. Together, these findings identify N218K as a novel regulator of HS-dependent attachment and CHIKV pathogenicity, reveal a high-attachment–low-dissemination phenotype, and identify enhanced post-attachment internalization as an additional phenotype associated with this substitution.

N218K fits a broader pattern established by previously reported CHIKV E2 mutations, in which changes in E2 charge can alter GAG utilization, viral dissemination, and pathogenicity [11,12,35]. Notably, sequence analysis showed that N218 is conserved among representative strains of the major CHIKV lineages examined, including the Asian, West African, ECSA, ECSA-IOL, and ECSA-2 lineages (Figure S3A), and is also conserved among the representative arthritogenic alphaviruses analyzed (Figure S3B). Whether N218K was already present in the original clinical sample or emerged or was selected during virus isolation and early passage in vitro cannot be determined from the present data. A previous study reported that the corresponding N218R substitution in RRV altered plaque morphology and enhanced HS-dependent infectivity [14,36], closely resembling the phenotype observed here for CHIKV N218K. Alteration at this conserved position affected viral attachment, systemic dissemination, and pathogenicity in a manner similar to that reported for RRV, while CHIKV N218K additionally revealed a previously unrecognized role for residue 218 in regulating viral internalization.

The enhanced attachment and restricted spread observed with N218K are not necessarily contradictory and may reflect different consequences of altered virus–cell surface interactions. Increased HS-dependent attachment can increase the probability of successful infection in cultured mammalian cells, consistent with the lower viral RNA-to-PFU ratio and higher infectious titers of rCHIKV-N218K. The absence of an attachment phenotype in C6/36 cells further indicates that the effect of N218K depends on the surface properties of the target cell, including the availability and characteristics of cell-surface GAGs. At the same time, restoration of N218K plaque size by DEAE-dextran supports a role for charge-dependent extracellular interactions in restricting local viral spread. A similar principle may explain why enhanced infection in vitro does not translate into greater pathogenicity in vivo. rCHIKV-N218K produced lower early viremia and was associated with lower tissue viral RNA burdens at 5 dpi. These findings are consistent with reduced systemic dissemination, although the magnitude of the tissue RNA differences at 5 dpi may partly reflect differences in disease stage. Previous studies have similarly linked strong GAG binding to attenuation of CHIKV and other alphaviruses, whereas weaker HS binding can prolong circulation and increase viremia [12,35]. Vascular GAGs and blood-filtering cells may capture strongly GAG-binding virions and accelerate their removal from the circulation [37,38]. Although viral clearance and vascular capture were not directly measured in this study, the combination of reduced early viremia, relatively modest differences in viral burden at the inoculation site, and more pronounced reductions in distal tissues and organs is consistent with reduced systemic dissemination. These observations are also compatible with, but do not directly establish, previously proposed attenuation mechanisms associated with enhanced GAG interactions.

The enhanced internalization of rCHIKV-N218K indicates that the effect of N218K extends beyond initial attachment to a post-attachment uptake step. HS has generally been considered to promote nonspecific electrostatic attachment of virions to the cell surface, thereby increasing the opportunity for subsequent engagement of protein receptors, but whether changes in HS-dependent attachment can also influence subsequent internalization remains less clear. However, recent work on EEEV has challenged this conventional view [39]. Positively charged HS-binding regions on E2 were found to overlap with protein receptor-binding regions and to contribute to both HS utilization and LDL receptor family-mediated infection, indicating that attachment-factor binding and protein receptor engagement can be functionally coupled. Importantly, N218K increased internalization in Vero and A549 cells even when the initial amount of cell-bound virus was controlled. This finding indicates that the effect of N218K is not limited to initial attachment but also affects a post-attachment internalization step. The present assay does not determine the receptor or cellular pathway responsible for this phenotype. The mechanism underlying this post-attachment effect remains to be determined.

Two limitations should be considered when interpreting the in vivo results. First, CHIKV-S also carries three nsP1 substitutions (Y157H, R171Q, and P476Q), and its overall phenotype therefore cannot be attributed entirely to N218K. The reverse-genetic experiments demonstrate that N218K alone is sufficient to reproduce the small-plaque phenotype in the parental infectious-clone background. However, possible contributions or epistatic effects of the three nsP1 substitutions on the early growth and in vivo phenotypes of CHIKV-S cannot be excluded. The functions of these nsP1 substitutions have been addressed in previous studies or are being investigated separately. Second, tissue samples were collected at 5 dpi, when disease progression had already begun to differ between parental rCHIKV and rCHIKV-N218K. The magnitude of the tissue viral RNA differences may therefore partly reflect differences in disease stage. The lower serum viral RNA levels at 1 and 3 dpi, before mortality occurred in either group, provide stronger evidence that N218K reduces early systemic dissemination.

In summary, E2 N218K is a previously unrecognized CHIKV determinant of HS-dependent attachment and partial attenuation in A129 mice, with an additional effect on post-attachment viral internalization. N218K enhances HS-dependent attachment and viral internalization in mammalian cells and is associated with reduced systemic dissemination in A129 mice, revealing a high-attachment–low-dissemination phenotype. Given the conservation of residue 218 among CHIKV lineages and arthritogenic alphaviruses, this site may warrant further evaluation as a component of attenuation strategies in combination with independent attenuating modifications.

## Data Availability

The data generated or analyzed during this study are available within the article and its Supplementary Materials.

## Supplementary Materials

The following supporting information can be downloaded at: https://www.mdpi.com/article/10.3390/pathogens15090993/s1, Figure S1. Stability and competitive advantage of the CHIKV-S small-plaque phenotype during serial passage; Figure S2. Genetic and phenotypic stability of E2 N218K during in vivo infection; Figure S3. Conservation of E2 residue N218 among CHIKV lineages and related arthritogenic alphaviruses; Table S1. Nucleotide variants identified by deep sequencing of the GD2010 CHIKV stock.
