# Supplementary material for: Chikungunya Virus E2 N218K Enhances Heparan Sulfate-Dependent Attachment and Viral Internalization While Conferring Partial Attenuation in A129 Mice
Source: Pathogens. 2026 Sep 19;15(9):993. doi: 10.3390/pathogens15090993 (PMC13610807; doi:10.3390/pathogens15090993)
Supplement: Supplementary file 1 [file pathogens-15-00993-s001.zip › pathogens-4522416-supplementary.pdf]

Supplementary materials

Table S1. Nucleotide variants identified by deep sequencing of the GD2010 CHIKV stock.

Genome position	Ref	Alt	Gene	Read counts (Alt:Ref)	Variant frequency (%)	Codon change	Amino acid change	Residue position
545	T	C	nsP1	C:7076 T:4647	60.36%	TAC→CAC	Y→H	157
588	G	A	nsP1	A:1268 G:10373	10.89%	CGA→CAA	R→Q	171
1502	C	A	nsP1	A:1644 C:5432	23.23%	CCA→CAA	P→Q	476
3397	T	C	nsP2	C:14620 T:0	100.00%	TAT→TAC	Y→Y	572
9195	T	A	E2	A:5232 T:8953	36.88%	AAT→AAA	N→K	218
11779	A	C	3'UTR	C:121 A:1632	6.90%	/	/	/
11781	G	C	3'UTR	C:111 G:1178	8.61%	/	/	/
11788	A	C	3'UTR	C:63 A:613	9.32%	/	/	/
11789	T	C	3'UTR	C:45 T:530	7.83%	/	/	/
11790	G	C	3'UTR	C:104 G:348	23.01%	/	/	/
11791	T	C	3'UTR	C:77 T:240	24.29%	/	/	/

Note: Genome positions are numbered according to the CHIKV GD2010 reference genome (GenBank accession no. JX088705.1). Ref and Alt indicate the reference and alternative nucleotides, respectively. Variant frequency was calculated as Alt/(Alt + Ref) × 100%. Amino acid positions are numbered within the indicated viral protein. Nonsynonymous substitutions are highlighted in red, whereas Y→Y denotes a synonymous substitution. A slash (/) indicates that the entry is not applicable. nsP1 and nsP2, nonstructural proteins 1 and 2, respectively; E2, envelope glycoprotein E2; 3′ UTR, 3′ untranslated region.

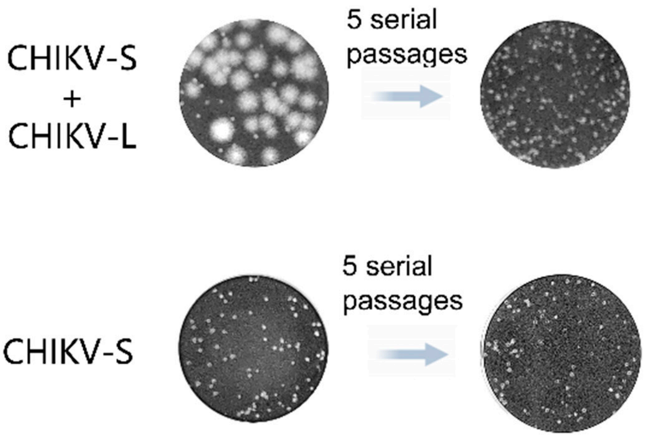

Figure S1. Stability and competitive advantage of the CHIKV-S small-plaque phenotype during serial passage. Equal PFU amounts of CHIKV-L and CHIKV-S were mixed and serially passaged in Vero cells for five passages (top). At passage 5, only the small-plaque phenotype was detectable. Plaque-purified CHIKV-S was independently serially passaged for five passages to assess phenotypic stability (bottom). Representative plaque morphologies before and after serial passage are shown.

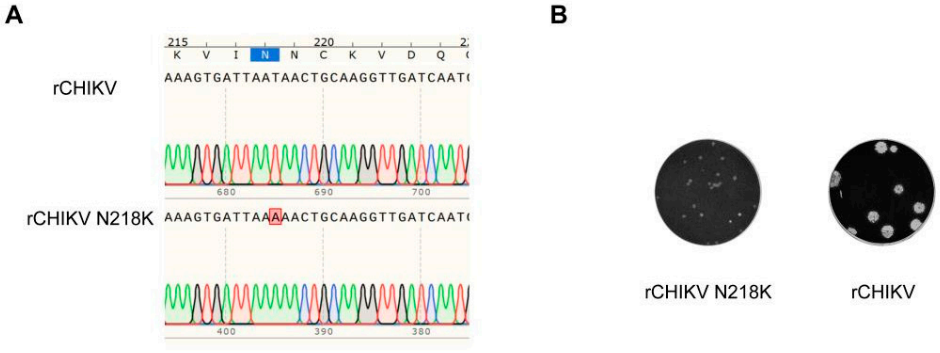

Figure S2. Genetic and phenotypic stability of E2 N218K during in vivo infection. (A) Sequence analysis of viruses recovered from joint tissues at 5 dpi confirmed retention of the expected E2-218 residue in rCHIKV and rCHIKV-N218K, with no detectable K-to-N reversion. Sequencing depths at residue 218 were 1,062 and 1,309 reads, respectively. (B) Representative plaque morphologies of viruses recovered from joint tissues at 5 dpi. rCHIKV-N218K retained the small-plaque phenotype, whereas parental rCHIKV retained

the large-plaque phenotype.

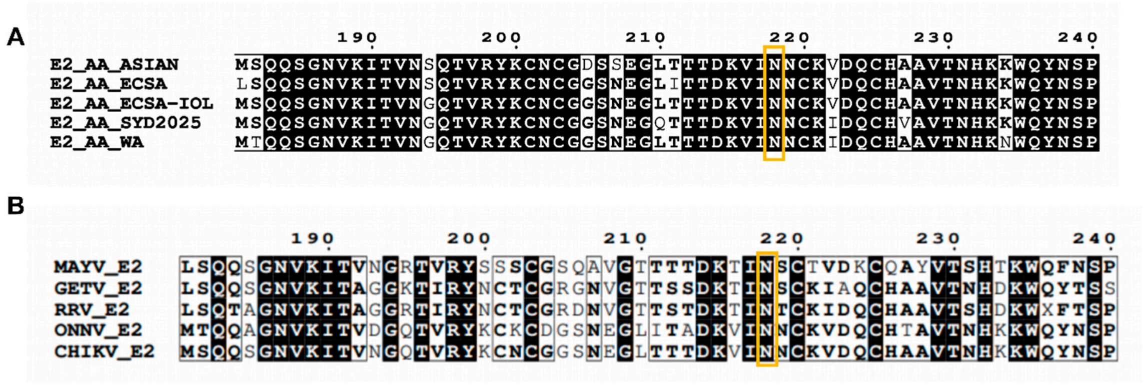

Figure S3. Conservation of E2 residue N218 among CHIKV lineages and related arthritogenic alphaviruses. **(A)** Full-length E2 amino acid sequences from representative strains of the Asian, East/Central/South African (ECSA), ECSA–Indian Ocean lineage (ECSA-IOL), ECSA-2, and West African (WA) lineages were aligned. SYD2025 was included as the representative strain of the ECSA-2 lineage. **(B)** Full-length E2 amino acid sequences of Mayaro virus (MAYV), Getah virus (GETV), Ross River virus (RRV), O'nyong-nyong virus (ONNV), and CHIKV were aligned. For clarity, only the region corresponding to CHIKV E2 residues 180–240 is shown. CHIKV residue N218 and the corresponding conserved asparagine residues are highlighted by yellow boxes. Identical residues are shown with black shading.
